# Rapid Estimation Method for Gear Bending Fatigue Limit

**DOI:** 10.3390/ma19173582

**Published:** 2026-08-24

**Authors:** Changshu Yang, Xinhao Zhao, Xiaofeng Yu, Zhengminqing Li, Wenqiu Li

**Affiliations:** 1 AECC Beijing Institute of Aeronautical Materials, Beijing 100095, China; 2 AECC Zhongchuan Transmission Machinery Co., Ltd., Changsha 410200, China; zhaoxh729@foxmail.com; 3 College of Mechanical and Electrical Engineering, Nanjing University of Aeronautics and Astronautics, Nanjing 210016, China; yxf2105511@nuaa.edu.cn; 4 School of Mechatronics Engineering, Henan University of Science and Technology, Luoyang 471003, China; liwenqiu@stu.haust.edu.cn

**Keywords:** gear fatigue limit, small samples, fatigue strength reduction factor

## Abstract

**Highlights:**

- A calculation method for rapid estimation of gear bending fatigue limit has been established.
- Based on the rapid test theory and the principle of small sample statistics, combined with the material’s tension–compression S-N curve and step-down loading method, the safe fatigue limit of gears under a certain reliability level is estimated.
- This method applies gears made from new processes and materials to accelerate process iteration.

**Abstract:**

The fatigue performance of gears is very important to the safe operation of the transmission system. For gears fabricated using novel materials and advanced manufacturing processes, fatigue limit testing entails significant costs and prolonged timeframes, thereby constraining the rate of design iteration. In this paper, a rapid prediction method for the bending fatigue limit of gears is proposed. This method starts with the S-N curve of the material. Based on the rapid test theory and the principle of small sample statistics, combined with the step-down loading method, the safety fatigue limit of the gear under certain reliability is estimated. Compared with the up-and-down method, the error of this method is within 7.8%, verifying the feasibility of this data processing. At the same time, in this paper, the bending fatigue test of corrosion-resistant nitrided steel gears is carried out, and the bending fatigue limit of corrosion-resistant nitrided steel gears under 99% reliability is quickly obtained. This method holds significant engineering significance in saving test costs, reducing the number of tests, and shortening the development cycle. However, it is currently only applicable to standard spur gears and does not yet consider factors such as the geometric characteristics of helical gears and the corrosion resistance of materials.

## 1. Introduction

Gears are fundamental components of transmission systems, and their fatigue performance significantly influences the product’s service life and reliability [1,2]. The fatigue limit of gears serves as a critical metric for assessing their fatigue performance, while conducting gear fatigue tests is the most direct and effective method for determining this limit. Currently, well-established international standards exist that define the test methodologies and data analysis approaches for gear fatigue experiments. The FVA guideline 563 I provides standardized recommendations for conducting flank and root load-carrying capacity tests on through-hardened and case-hardened cylindrical gears, covering material selection, specimen manufacturing, heat treatment, quality control, test procedures, and statistical evaluation [3]. ISO 12107:2012 provides statistical methods for the planning of fatigue tests and the analysis of resulting data, including the estimation of fatigue life at a given stress, fatigue strength at a given life, and the S-N curve with confidence and tolerance limits [4]. In testing, the up-and-down method is typically employed to obtain the fatigue limit of gears [5,6]. To ensure the accuracy and reliability of test results, the conventional up-and-down method requires a minimum of 20 valid data points. This requirement necessitates extensive testing with numerous specimens, leading to prolonged continuous monitoring and considerable resource investment, which presents a significant cost challenge [7]. For gears utilizing novel processes and materials, where both the manufacturing process and fatigue performance are still under exploration, allocating substantial resources to fatigue limit testing is clearly not cost-effective [8]. Therefore, during the initial phases of implementing new processes and materials, identifying a more efficient and economical testing or prediction method is crucial for evaluating fatigue performance and designing for reliability [9,10]. Fu Huimin [11] et al. assumed that fatigue life follows a log-normal distribution and employed heteroscedastic regression analysis to conduct a comprehensive analysis of fatigue test data. Xie et al. [12] converted fatigue lives tested at different stress levels into equivalent lives at any stress level, effectively transforming small sample data into large-scale samples and deriving the P-S-N curve for gears. Gao et al. [13], based on the premise that P-S-N curves intersect at a specific point in the high-stress region, successfully expedited the acquisition of P-S-N curves.

With the advancement of machine learning, artificial intelligence, and related technologies, these methods are increasingly being applied to research on fatigue life prediction [14,15,16]. However, gear fatigue research requires a large number of experiments for verification, and the application of these methods to the study of new material gear processes is time-consuming and costly. To enable rapid and effective estimation of the fatigue limit of gears using a single test specimen, a method for quickly estimating the gear fatigue limit based on the material’s tensile–compressive S-N curve is proposed in this paper. Starting from the material’s tensile–compressive S-N curve, this method leverages the commonality between material and gear fatigue failure mechanisms, employs the step-down loading method to conduct gear bending fatigue tests, accounts for variability in the gear manufacturing process, and integrates small-sample statistical principles to calculate the safe fatigue limit of gears at a specific reliability level. Since corrosion-resistant nitrided steel is a new type of material and belongs to the third generation of aviation gear steel, there is currently little data on S-N curves for gear materials and processes that are identical or similar to those of corrosion-resistant nitrided steel. Therefore, this paper proposes using the tensile–compressive S-N curve of corrosion-resistant nitrided steel material, combined with the step-down loading method, to quickly estimate the fatigue limit of gears.

## 2. Gear Fatigue Limit Test Estimation Method

### 2.1. Method for Calculating Average Fatigue Limit

According to Bayes’ theory [17] and the accelerated life testing theory [18], the fatigue characteristics of gears conform to the following three assumptions:

The standard specimen of gear steel exhibits a uniform stress distribution across its cross-section under axial tensile and compressive loads; however, under bending loads, the stress follows a linear distribution pattern along the height of the cross-section, with the maximum tensile/compressive stresses concentrated at the specimen’s surface. Due to the stress gradient and volume effect, the bending fatigue strength is generally higher than the tensile–compressive fatigue strength, and their equivalent relationship is shown in Equation (1).

(1) $$\sigma_{b}={\sigma_{a}\cdot K}_{f}$$

where $\sigma_{b}$ is the stress amplitude under bending load; $\sigma_{a}$ is the stress amplitude under tensile–compressive load; and $K_{f}$ is the fatigue strength correction factor.

As critical transmission components, gears exhibit high-cycle fatigue characteristics, and their fatigue properties can be reasonably characterized using either two-parameter or three-parameter S-N curve equations. Nevertheless, the three-parameter S-N curve can describe the fatigue damage accumulation laws of gears from the low-cycle fatigue life region, through the finite fatigue life region, to the high-cycle fatigue life region, providing a more comprehensive description of the fatigue characteristics of gears. It is suitable for predicting the fatigue limit under different stress levels [19]. Therefore, the three-parameter S-N curve equation of standard specimens is obtained through uniaxial tension–compression fatigue tests of gear steel standard specimens. Since the load can be equivalently processed, the S-N curve of the gear has the same form as the S-N curve of the standard specimen, as shown in Equation (2).

(2) $$(\sigma_{s}-\sigma_{a\infty }{)}^{m}\times N=C$$

In the equation, *m* and *C* are shape parameters determined through experiments; *σ_s_* represents the cyclic stress level applied to the standard specimen; *N* denotes the fatigue life at a given stress level; and σ*_a∞_* signifies the fatigue limit stress in the infinite life region.

When considering only a single gear, it can be assumed that the dispersion pattern of life under different reliability levels is consistent. Therefore, it is hypothesized that the S-N curves for the gear at various reliability levels share the same shape parameter, which can be expressed by Equation (3).

(3) $$(\sigma_{i}-\sigma_{R,\infty }{)}^{m}\times N_{i}=C$$

In the equation, $\sigma_{i}$ represents the test stress level, and $N_{i}$ denotes the number of fatigue cycles corresponding to the stress level $\sigma_{i}$ at level i. Based on the fatigue test data of the gear and in conjunction with the Miner’s linear cumulative damage theory as shown in the equation, $\sigma_{R,\infty }$ is calculated and determined as illustrated in Equation (4).

(4) $$\sum_{i=1}^{n}\frac{n_{i}}{N_{i}}=1$$

Assuming the fatigue limit stress of the gear is a statistical variable, and σ or lnσ follows a normal distribution, by employing the statistical analysis method of the normal distribution, the fatigue limit of the gear with a specific reliability can be obtained.

Through uniaxial tension–compression tests on standard gear steel specimens, the S-N curve of the gear steel specimen is obtained, and further, the equivalent form of the S-N curve equation for the gear is derived. Subsequently, gear fatigue tests are carried out using the accelerated life testing theory, where the stress levels in the tests are increased to accelerate the fatigue testing process, thereby rapidly obtaining fatigue test data within a short period. Based on the fatigue test data, the three-parameter S-N curve of the gear is combined with the cumulative damage theory. By applying the corresponding equations to process the test data, the mean fatigue limit $\sigma_{0.5,\infty }$ of the gear can be ultimately determined as the safe fatigue limit with a certain reliability. Furthermore, based on the principles of small-sample mathematical statistics, the sample mean and standard deviation are calculated.

### 2.2. Fatigue Strength Reduction Factor Calculation Method

The results of gear fatigue limit tests often exhibit significant variability due to errors in material properties, heat treatment, processing, and loading. Therefore, based on the principles of mathematical statistics and assuming that the fatigue limit stress follows a log-normal distribution, the average fatigue limit is divided by a fatigue strength reduction factor *J_R_* with a certain reliability to determine the safe fatigue limit of the gear, taking into account the variability introduced by material properties, heat treatment processes, processing, and loading errors in the gear test specimens.

There are two methods for calculating the fatigue strength reduction factor: the one-sided tolerance coefficient method and the comprehensive standard deviation method [20]. Currently, the one-sided tolerance coefficient method is commonly used to calculate the fatigue strength reduction factor. However, the safe fatigue strength value determined by this method deviates significantly from the sample mean, and boundary effects occur when the number of test specimens is small, resulting in negative one-sided tolerance coefficient values. Therefore, the one-sided tolerance coefficient method is not suitable for cases with small sample sizes. The comprehensive standard deviation method determines the tolerance lower limit by fully utilizing the data information of low-carbon steel, treating the current test sample as a small sample of a sample set (approximate population), and using the concept of comprehensive standard deviation to make the estimated fatigue limit result closer to the population. Moreover, the one-sided tolerance factor value calculated by the comprehensive standard deviation method remains relatively stable as the number of samples decreases, and it can satisfactorily estimate the population’s safe fatigue strength even when the sample size is as small as one [19].

As known from mathematical statistics, the lower confidence limit with confidence level r for the percentile position of population X is shown in Equation (5).

(5) $$P\left\{X+\left(u_{R}-u_{r}/\;\sqrt{n}\right)\sigma \;\leq X_{R}\right\}=r$$

In the equation, the population standard deviation σ is unknown, making it impossible to determine this confidence lower limit. However, by referring to similar data or past experience, it can be concluded that the population standard deviation will not exceed or, with a certain confidence level, be less than a certain value $\hat{\sigma }$, referred to as the combined standard deviation, as shown in Equation (6).

(6) $$P\left(\sigma \;\leq \hat{\sigma }\right)=r_{\sigma }$$

In the equation, $r_{\sigma }$ represents the confidence level that the true value σ is less than the combined standard deviation $\hat{\sigma }$, typically set at 90% or 95%. The lower confidence limit of the safety strength ${\hat{X}}_{R}$ can then be calculated according to Equations (7) and (8).

(7) $${\hat{X}}_{R}=X+(u_{R}-u_{r}/\sqrt{n})\hat{\sigma }$$

(8) $$P\left({\hat{X}}_{R}\leq X_{R}\right)=r$$

The fatigue limit of gears follows a log-normal distribution, i.e., $ln\sigma_{\infty }\sim \left(\mu \;,\;\sigma^{2}\right)$, and then the mean $ln\sigma_{0.5,\infty }$ and the sample standard deviation s of the test samples can be expressed by Equation (9).

(9) $$\ln\sigma_{0.5,\infty }=\frac{1}{n}\sum_{i=1}^{n}ln\sigma_{i\infty }$$

(10) $$s=\sqrt{\frac{\sum_{i=1}^{n}{\left(ln\sigma_{i\infty }-ln\sigma_{0.5,\infty }\right)}^{2}}{n-1}}$$

In the equation, $\sigma_{i\infty }$ represents the fatigue limit of the *i*-th test specimen.

The fatigue strength reduction factor is defined as the ratio of the mean fatigue limit $\sigma_{0.5,\infty }$ to the safe fatigue limit $\sigma_{R}{,}_{\infty }$ at a reliability level of R, according to the comprehensive standard deviation method, expressed by Equation (11).

(11) $$J_{R}=\frac{\sigma_{0.5,\infty }}{\sigma_{R,\infty }}$$

The fatigue strength scatter factor is calculated using the comprehensive standard deviation method as shown in Equation (12).

(12) $$J_{R}=e^{(\mu_{r}/\sqrt{n}-\mu_{R})\sigma }$$

In the formula, $\mu_{r}$ is the standard normal deviate at a certain confidence level, and $\mu_{R}$ is the standard normal deviate at a certain reliability level, which can be checked in the normal distribution table; n is the number of test specimens or the sample size; and σ is the population standard deviation of the fatigue limit, which is a statistical measure used to describe the degree of dispersion of material property parameters. Statistical data indicate that the population standard deviation of the fatigue strength of low-carbon steel is 6% [19].

### 2.3. Method Feasibility Verification

Using the aforementioned data processing method, the bending fatigue limit of the 18CrNiMo7-6 gear at 3 × 10^6^ cycles is estimated based on the continuous incremental load test data of a single point from reference [21]. This estimation is then compared with the bending fatigue limit obtained from the step-down loading method using 20 valid data points at the same number of cycles from reference [22].

The bending fatigue limits of the 18CrNiMo7-6 gear with a module of 12 mm, obtained through the tooth number variation method and the minimal point test method, are shown in Table 1.

As shown in Table 1, compared with the step-up and step-down method, the bending fatigue limit value obtained by the data processing method proposed in this paper is more conservative, with an error of 3.3% at 50% reliability and 7.8% at 99% reliability, and the error is within 7.8%. This verifies the feasibility of this experimental data processing method for estimating gear fatigue limits, indicating its potential application value in quickly and cost-effectively estimating the fatigue limits of gears made from new materials and processes.

## 3. Bending Fatigue Test of Corrosion-Resistant Nitrided Steel Gears

According to the estimation method of gear fatigue limit, the bending fatigue test of corrosion-resistant nitrided steel gears was conducted using the step-down loading method, and the test data were processed. The test employed a pulsating loading mode and was performed on a high-frequency fatigue testing machine using a proprietary fixture until the gear teeth experienced bending fatigue failure, thereby obtaining the test data for bending fatigue damage. The criteria for gear bending failure are as follows: fracture of the gear at the tooth root; a reduction in test frequency by 5–10%; and confirmation of cracks upon shutdown.

### 3.1. Test Conditions

**Figure 1:**
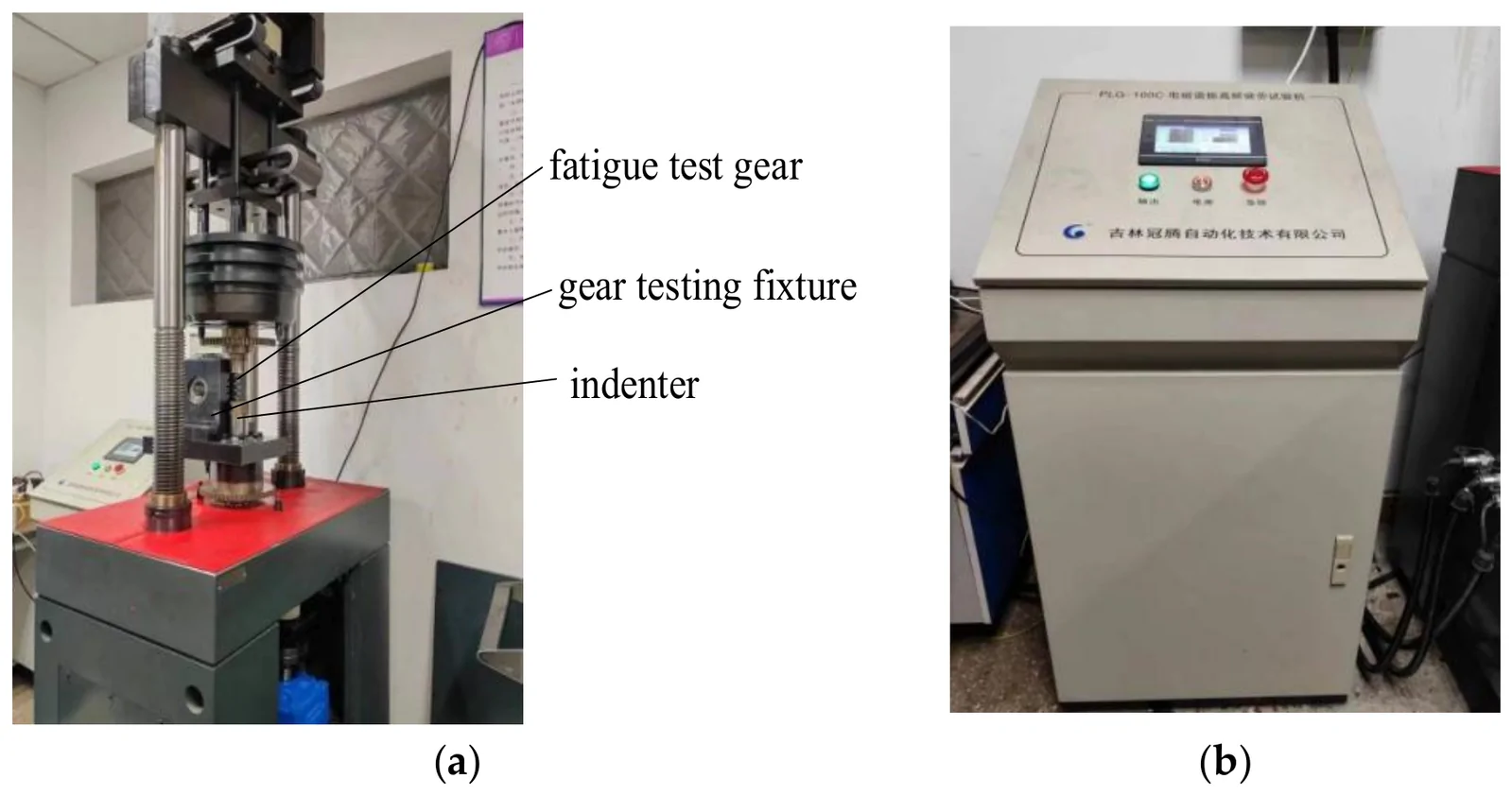
PLG-100C high frequency fatigue testing machine. (**a**) Experimental equipment. (**b**) Control equipment.

Test Equipment: The test employs the PLG-100C high-frequency fatigue testing machine (Jilin Guanteng Automation Technology Co., Ltd., Changchun, China), with a maximum static load of ±100 kN, an adjustable load of 50 kN, and a maximum frequency of 250 Hz. It is equipped with display and recording devices for static and dynamic loads, loading frequency, and cycle count, with an error not exceeding ±0.1%. Within the loading range of 5% to 100%, the relative error of the static load indication does not exceed ±1%, and the relative error of the dynamic load indication does not exceed ±3%. The specific test equipment is shown in Figure 1: Figure 1a shows the experimental equipment, including fatigue test gear, indenter, etc., and Figure 1b is the control equipment.

Test Specimen: The test gears are manufactured from corrosion-resistant nitriding steel, and the specific geometric parameters of the gears are listed in Table 2.

**Figure 2:**
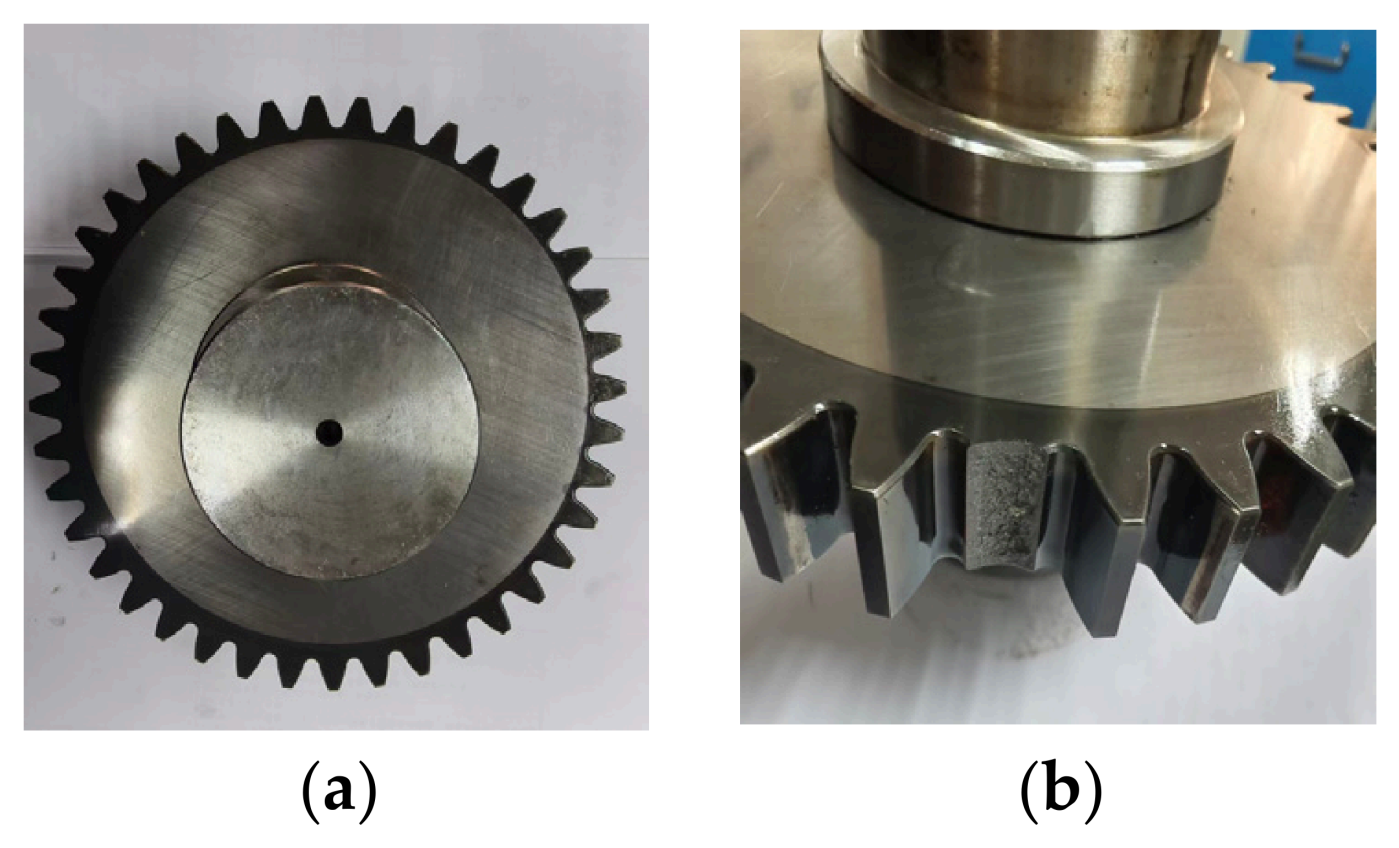
Bending test gear. (**a**) Test gear. (**b**) Failed gear.

The gear manufacturing process flow is as follows: forging–normalizing–rough turning–quenching and tempering–semi-finish turning–gear hobbing–solution nitriding–quenching–cryogenic treatment–tempering–gear grinding–shot peening. The surface hardness of the tooth root is 710~735 HV, with an effective hardening depth of 1.4~1.6 mm. The average residual stress at the reference circle of the tooth surface is −1230.5 MPa, and the average residual stress at the tooth root fillet is −1012 MPa. The physical image of the gear is shown in Figure 2. Figure 2a shows the test gear, and Figure 2b shows the failed gear.

**Figure 3:**
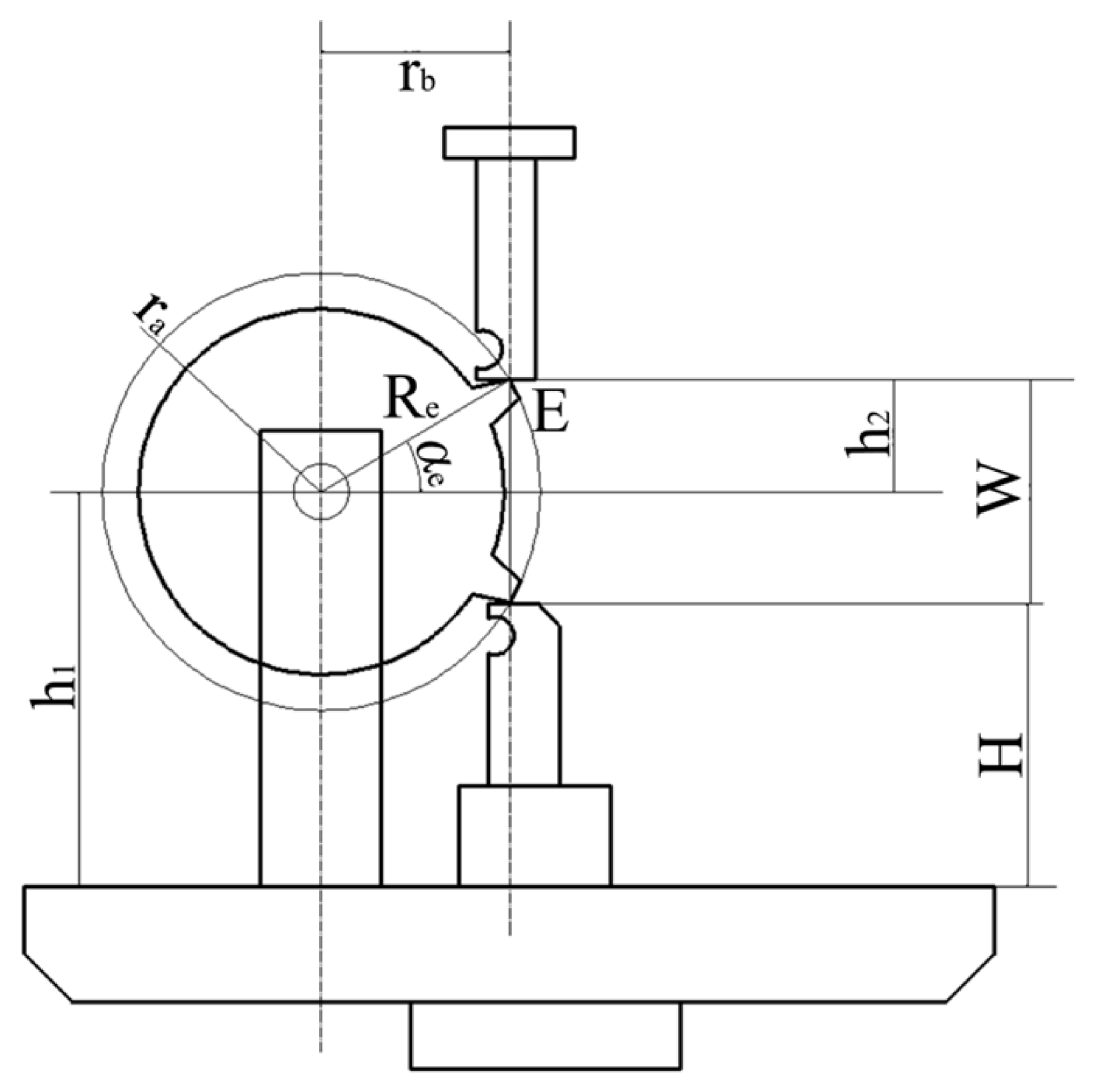
Fixture diagram of bending fatigue test.

In the pulsating bending fatigue test, the principle for correctly clamping the gear is as follows: The horizontal plane of the indenter is tangent to the tooth profile at the loading point, and the line of action of the loading force is tangent to the base circle of the gear. This method requires designing a dedicated fixture according to the national standard GB/T 14230 [23]. The schematic diagram of the fixture is shown in Figure 3, and the fixture parameters are listed in Table 3. During the test, the pulsating load is applied only to a single tooth symmetrical to the test tooth. The test gear does not engage in operation. To minimize the impact of the test tooth on the fatigue life of adjacent teeth, the selected test tooth should be at least one tooth apart from the loading tooth (including the supporting tooth).

Figure 3 shows the fixture diagram of bending fatigue test, in which H is the height of the supporting point of the supporting tooth; W is the common normal length of the overload point *E*; R_E_ is the radius of the circle where the load acts on point *E*; h_1_ is the vertical height from the load point to the center of the test gear; and h_2_ is the vertical height from the load point to the center of the test gear.

### 3.2. Experimental Data and Processing

Using 728 MPa as the initial stress of the gear, a bending fatigue test was conducted by applying stepwise loading on a single tooth, with each stress level cycled 1 × 10^6^ times. The test loading was calculated according to Equation (13) to determine the corresponding root bending stress, and the test data obtained is shown in Table 4.

(13) $${\sigma^{\prime }}_{F}=\frac{F_{t}\cdot Y_{FE}\cdot Y_{SE}}{b\cdot m\cdot Y_{ST}\cdot Y_{\delta relT}\cdot Y_{RrelT}\cdot Y_{X}}$$

In the formula, $F_{t}$ is the nominal tangential force; b is the tooth width; m is the module; $Y_{FE}$ is the tooth form factor when the load is applied to the tooth; $Y_{SE}$ is the stress correction factor when the load is applied to the tooth; $Y_{ST}$ is the stress correction factor for the test gear; $Y_{\delta relT}$ is the relative root fillet sensitivity factor; $Y_{RrelT}$ is the relative root surface condition factor; and $Y_{X}$ is the size factor for bending strength calculation.

According to the data in Table 4, it can be seen that there was no failure at stress levels of 728, 752, 778, and 803 MPa after 1 × 10^6^ cycles, while cracks occurred at a stress of 828 MPa after 2.54 × 10^4^ cycles.

Using smooth test bars made of corrosion-resistant nitrided steel with a diameter of 7 mm and Kt=1, axial tensile fatigue tests were conducted under the stress ratio r=0. The fatigue life test results for each stress level of the test bars are shown in Table 5.

According to Table 5, the sample can basically withstand 1 × 10^7^ cycles at 980 MPa, while at 1200 MPa the number of cycles is about 10^4^.

The logarithm of both sides of the three-parameter S-N curve (2) is taken, and the three-parameter S-N curve for corrosion-resistant nitrided steel specimens is fitted based on the data in Table 5, as shown in Equation (14).

(14) $$m\ln(\sigma_{s}-\sigma_{\infty })+\ln N=\ln C$$

Let $x=ln\left(\sigma_{s}-\sigma_{\infty }\right)$, y=lnN, a=lnC, and b=−m; the above equation can be transformed into the form y=a+bx, and by the method of least squares, we obtain

(15) $$\left\{\begin{array}{c} b=L_{xy}/L_{xx}\\ a=\bar{y}-\bar{x}b \end{array}\right.$$

In the formula,

(16) $$\left\{\begin{array}{c} L_{xx}=\sum_{i=1}^{n}x_{i}^{2}-\frac{1}{n}{\left(\sum_{i=1}^{n}x_{i}\right)}^{2}\\ L_{yy}=\sum_{i=1}^{n}y_{i}^{2}-\frac{1}{n}{\left(\sum_{i=1}^{n}y_{i}\right)}^{2}\\ L_{xy}=\sum_{i=1}^{n}x_{i}y_{i}-\frac{1}{n}\left(\sum_{i=1}^{n}x_{i}\right)\left(\sum_{i=1}^{n}y_{i}\right) \end{array}\right.$$

The square of the linear correlation coefficient R is

(17) $$R^{2}=\frac{L_{xy}^{2}}{L_{xx}L_{yy}}$$

Since $\sigma_{\infty }$ is unknown, Equation (16) can be regarded as a function of $\sigma_{\infty }$. When the absolute value of the linear correlation coefficient R reaches its maximum, the fitted equation exhibits the best correlation. It is derived as Equation (18), and by finding the extremum of R, $\sigma_{\infty }$ can be determined.

(18) $$\frac{d(R^{2})}{d\sigma_{\infty }}=0$$

In the formula,

(19) $$\frac{d(R^{2})}{d\sigma_{\infty }}=\frac{L_{xy}^{2}}{L_{xx}L_{yy}}\left(\frac{2}{L_{xy}}\frac{dL_{xy}}{d\sigma_{\infty }}-\frac{1}{L_{xx}}\frac{dL_{xx}}{d\sigma_{\infty }}\right)$$

In Equation (20),

(20) $$\left\{\begin{array}{c} \frac{dL_{xy}}{d\sigma_{\infty }}=\sum_{i=1}^{n}\frac{y_{i}}{\sigma_{i}-\sigma_{\infty }}-\frac{1}{n}\left(\sum_{i=1}^{n}y_{i}\right)\left(\sum_{i=1}^{n}\frac{1}{\sigma_{i}-\sigma_{\infty }}\right)\\ \begin{array}{c} \frac{dL_{xx}}{d\sigma_{\infty }}=\sum_{i=1}^{n}\frac{x_{i}}{\sigma_{i}-\sigma_{\infty }}-\frac{1}{n}\left(\sum_{i=1}^{n}x_{i}\right)\left(\sum_{i=1}^{n}\frac{1}{\sigma_{i}-\sigma_{\infty }}\right)\\ \frac{dL_{xy}}{d\sigma_{\infty }}=L_{y0},\frac{dL_{xx}}{d\sigma_{\infty }}=L_{x0} \end{array} \end{array}\right.$$

Substituting Equations (17)–(19) yields the function of $\sigma_{\infty }$, as shown in Equation (20). Solving this Equation allows for the determination of $\sigma_{\infty }$.

(21) $$H\left(\sigma_{\infty }\right)=\frac{L_{y0}}{L_{xy}}-\frac{L_{x0}}{L_{xx}}=0$$

**Figure 4:**
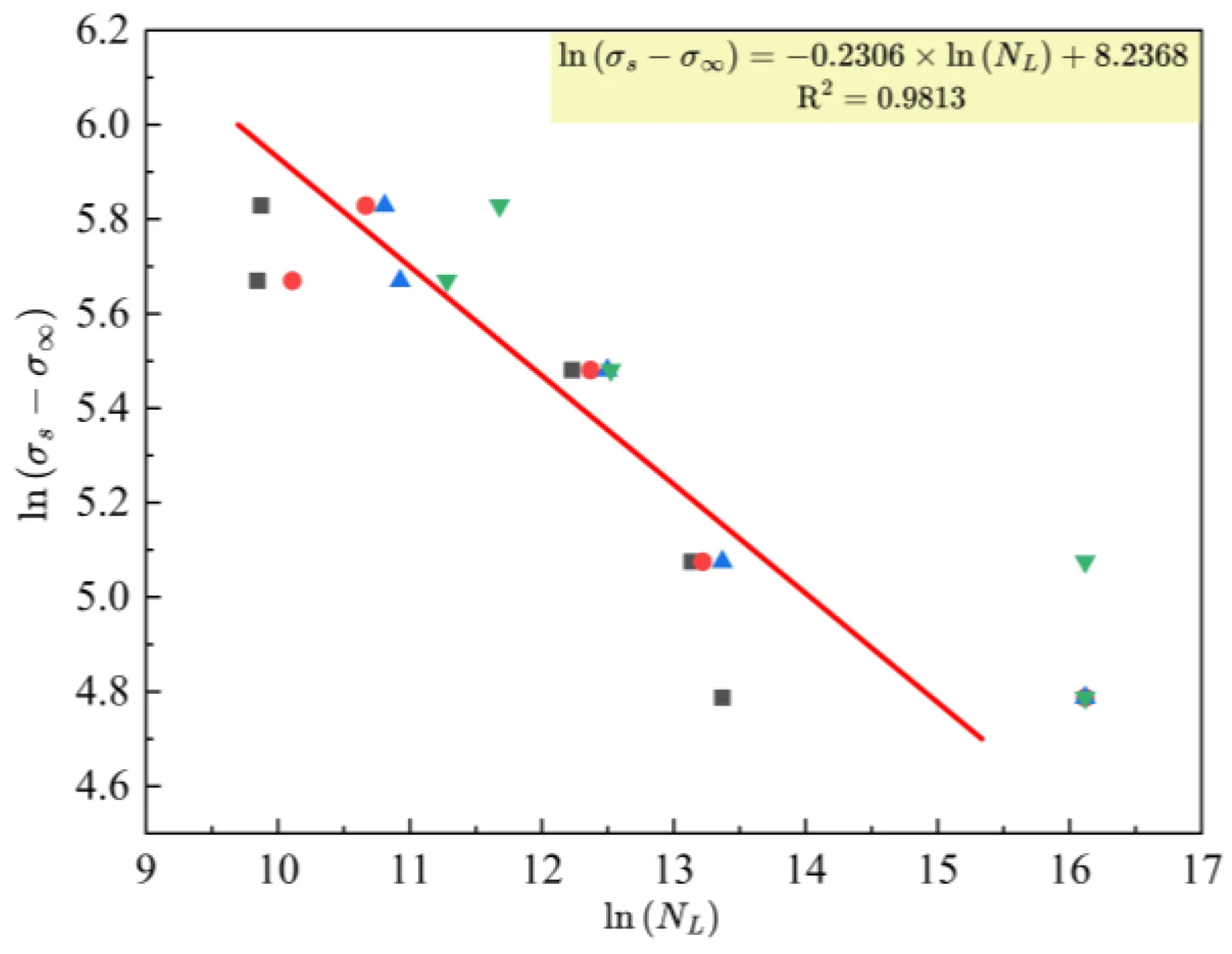
Three-parameter S-N curve of corrosion-resistant nitride steel test bar.

By further substituting σ_∞_ into Equation (20) and utilizing the least squares method to solve for the equation parameters *m* and *C*, the average fatigue S-N curve of the corrosion-resistant nitrided steel test bars is ultimately obtained, as illustrated in Figure 4.

*N_L_* is the fatigue life (number of cycles) of the material bar. As can be seen from the figure, the shape coefficient of the S-N curve is m = 4.3359, C = e^35.7140^. Based on the previous assumption that the S-N curves of the corrosion-resistant nitrided steel gear and the test specimen have the same form, the three-parameter bending S-N curve equation for the corrosion-resistant nitrided steel gear can be derived as shown in Equation (22).

(22) $$\ln N_{i}=-4.3359\ln(\sigma_{i}-\sigma_{0.5,\infty })+35.714$$

Based on the gear bending fatigue test data, the linear cumulative damage rule was applied to linearly superimpose the damage $D_{i}$ of the gear teeth under different stress levels. $D_{i}$ is defined as $D_{i}=\;n_{i}/N_{i}$. When the gear teeth experience bending fatigue failure at the tooth root, i.e., when the total damage reaches 1, the average fatigue limit $\sigma_{0.5,\infty }$ of the corrosion-resistant nitrided steel gear is determined to be 727.77 MPa. Substituting this value into the three-parameter S-N curve equation, Equation (21), for the gear, the average fatigue limit of the gear at the target number of cycles can be calculated from the curve. Through calculation, the average fatigue limit of the corrosion-resistant nitrided steel gear at 1 × 10^7^ cycles was determined to be 765.19 MPa.

### 3.3. Safety Fatigue Limit Estimation

Safety fatigue limit estimation is performed by considering the statistical dispersion of gear material properties and operating loads. Using the comprehensive standard deviation method, the strength reduction factor corresponding to 99% reliability is determined. The standard normal deviate at 99% reliability is −4.753, and the population standard deviation of commonly used gear steel is 6%. From Equation (7), the fatigue strength reduction factor JR at 99% reliability was calculated to be 1.175. According to the definition of the fatigue strength reduction factor, it is the ratio of the average fatigue limit to the fatigue limit at 99% reliability. Based on this calculation, the fatigue limit of corrosion-resistant nitrided steel gears at 99% reliability for 1 × 10^7^ cycles was determined to be 650.98 MPa.

## 4. Conclusions

A test method for estimating the fatigue limit of corrosion-resistant nitrided steel gears is established in this paper, and bending fatigue limit tests are conducted, with the following conclusions drawn.

(1) Based on the accelerated life theory and the mathematical statistical principles of small samples, and combined with the material’s S-N curve, it is possible to rapidly obtain a reliable gear bending safety fatigue limit value within a limited number of data points. The fatigue limit value obtained by this method is more conservative compared to the step-down loading method, with an error within 7.8%, demonstrating that this method has certain feasibility for gear bending fatigue limit estimation.

(2) By utilizing the three-parameter S-N curve of corrosion-resistant nitrided steel material, the S-N curve of the gear made from this material was obtained. Bending fatigue tests were conducted on the corrosion-resistant nitrided steel gears. Considering the dispersion of the gears, the comprehensive standard deviation method was employed to calculate the strength reduction factor under 99% reliability, thereby determining that the bending fatigue limit of the gear under 99% reliability is 650.98 MPa.

(3) For gears utilizing new processes and materials, the application of this testing estimation method enables rapid prediction of bending fatigue limits, which is of significant importance for accelerating process iteration. It should be noted that the application of this method to multiaxial loading scenarios (e.g., rolling contact conditions of gears) requires further verification through additional tests and model modifications.

## Figures and Tables

**Table 1:** Bending fatigue limit of 18CrNiMo7-6 gear under lifting method and small sample method.

	Step-Up and Step-Down Method	Method in This Paper	The Error
50% reliability	639 MPa	618 MPa	3.3%
99% reliability	603 MPa	556 MPa	7.8%

**Table 2:** Basic parameters of test gears.

Name	Value
Module/mm	4
Number of teeth/-	40
Pressure angle/(°)	20
Helix angle/(°)	0
Tooth width/mm	17
Tip diameter/mm	168
Base diameter/mm	150.351

**Table 3:** Basic parameters of fixture.

H/mm	W/mm	R_E_/mm	h_1_/mm	h_2_/mm
111	67	84	141	37

**Table 4:** Test fixture parameters.

Stress Level i	Root Bending Stress $\sigma_{i}$	Number of Cycles n
1	728	1 × 10^6^
2	752	1 × 10^6^
3	778	1 × 10^6^
4	803	1 × 10^6^
5	828	2.54 × 10^5^

**Table 5:** The life of each stress level of the test rod.

Stress Level MPa	Test Data 1	Test Data 2	Test Data 3	Test Data 4	Standard Deviation
980	639,192	10,001,003	10,001,003	10,001,006	4,680,906
1020	506,432	549,781	639,192	10,001,004	4,718,258
1100	204,465	235,462	267,480	274,360	32,148
1150	18,848	24,560	55,572	79,212	28,184
1200	19,358	42,831	49,392	118,203	42,507

## Data Availability

The original contributions presented in this study are included in the article. Further inquiries can be directed to the corresponding author.
